# Alleviation of postharvest chilling injury in sweet pepper using Salicylic acid foliar spraying incorporated with caraway oil coating under cold storage

**DOI:** 10.3389/fpls.2022.999518

**Published:** 2022-09-08

**Authors:** Saeed Hanaei, Hojatollah Bodaghi, Ziba Ghasimi Hagh

**Affiliations:** Department of Horticulture Science and Plant Protection, College of Agriculture, Shahrood University of Technology, Shahrood, Iran

**Keywords:** Salicylic acid foliar spraying, caraway oil-coating, cold storage, sweet pepper, chilling injury

## Abstract

The decrease in the postharvest quality of sweet peppers in terms of the physiological disorders resulting from cold storage (<7–10°C) results in the significant economic losses. The ability of pre-harvest foliar spraying of Salicylic acid (SA) (1.5 and 3 mM) and the postharvest caraway (*Carum carvi*) oil coating (0.3% and 0.6%) on chilling injury (CI) and the quality of stored sweet pepper at 4 ± 2°C for 60 d followed by an additional 2 d at 20°C were investigated. The antifungal activity of caraway oil (0.15%, 0.3%, and 0.6%) on *Botrytis cinerea* mycelia in *in vitro* showed that the maximum percentage of inhibition was equal to 95% in the medium with 0.6% of this oil. The CI of sweet pepper was significantly reduced by increasing SA, and caraway oil concentrations compared to the control, especially the lowest CI (14.36%), were obtained at 3 mM SA and 0.6% caraway oil treatment. The results showed a significant delay in the changes of weight loss (79.43%), firmness (30%), pH (6%), total soluble solids (TSS) (17%), titratable acidity (TA) (32%), and color surface characteristics and capsaicin content (5%) compared to control fruits at 3 mM SA and 0.6% caraway oil concentrations. Results indicated that the decrease in CI was related to a decrease in electrolyte leakage, malondialdehyde (MDA) content, total phenolic production, decay incidence, and an increase in the activity of antioxidant enzymes, including catalase (CAT), superoxide dismutase (SOD), and peroxidase (POD). Thus, the incorporation of SA (3 mM) and caraway oil (0.6%) to reduce the CI of stored sweet pepper at low temperature can be considered a practical solution to improve the quality and marketability of this product.

## Introduction

Sweet pepper is an important fruit crop in tropical and subtropical regions of the world and the greenhouse industry. Sweet pepper fruit is considered a suitable source of antioxidants, vitamins, carotenoids, flavonoids, phenolic acids, and other phytochemicals (Marín et al., 2004). Thus, the pepper fruits are currently introduced to prevent the certain types of cancer, cardiovascular disease, atherosclerosis, and suspension of the aging process (Simonne et al., 1997). The major factors for postharvest loss and quality deterioration of sweet pepper include water loss, chilling injury (CI), and pathological deterioration caused by *Botrytis cinerea* (Cheema et al., 2018).

Low-temperature storage is a prevalent method to extend fruits and vegetables' storage life and postharvest quality (Li et al., 2016). The sweet pepper fruits, however, are very susceptible to CI. CI occurs at a temperature below 7–10°C depending on the cultivar, maturity stage, and the duration of storage at low temperatures (Wang et al., 2016). Most studies on the alleviation of CI have focused on applying the modified atmosphere (Singh et al., 2014), UV-C (Vicente et al., 2005), hot water treatment (González-Aguilar et al., 2000), and the chemical materials like diphenylamine (Purvis, 2002), 5-aminolevulinic acid (Korkmaz et al., 2010), salicylate, jasmonates (Fung et al., 2004), and glycine betaine (Wang et al., 2016) during the postharvest storage. Recently, the effect of hot water combined with methyl salicylate (Rehman et al., 2021) and glutation (Yao et al., 2021) on CI reduction in sweet pepper has been studied. Furthermore, Wang et al. (2022) reported that the cold shock and oxalic acid are effective in CI reduction by increasing antioxidant enzyme activity and regulating proline metabolism (Wang et al., 2022).

As an endogenous signal molecule, Salicylic acid is essential for the plant's defense mechanism against oxidative stress because it promotes the production of antioxidant enzymes (Luo et al., 2011). SA induced the activity of β-1, 3-glucanase, phenylalanine ammonia-lyase (PAL), and POD during the short storage period in SA-treated cherries (Yao and Tian, 2005). It regulates some processes in the plants and interacts with the biosynthesis or action of ethylene, abscisic acid (ABA), and cytokinins (Srivastava and Dwivedi, 2000). Applying the postharvest SA and CaCl_2_ treatments delayed the ripening process of sweet pepper fruit and extended the shelf life of stored fruit at 10°C (Rao et al., 2011). An increase in the alternative oxidase (AOX) transcript using SA and MeSA before cold treatment resulted in a reduced incidence of CI in green bell peppers (Fung et al., 2004).

Furthermore, the postharvest semipermeable coating treatments were surveyed to maintain the quality of fruit and vegetables (Ojagh et al., 2010). Their barrier properties against solute, vapor, and gases reduce the moisture loss and respiration rate by slowing oxygen uptake. The chitosan coating enriched with cinnamon showed the higher activity of scavenger antioxidant enzymes including SOD, POD, and CAT in treated peppers during the 35 d of storage at 8°C (Xing et al., 2011).

Caraway (*Carum carvi*) is an endemic Iranian medicinal and aromatic plant (called “zireh siyah”) in the Apiaceae family, which possesses actively biochemical components such as carvone and limonene. It has the potential to substitute the conventional preservatives in food, cosmetic, and pharmaceutical industries (Trifan et al., 2016). Caraway oil was studied for its antifungal activities against *Penicillium digitatum* on orange fruits, and the results demonstrated positive effects of this oil on storage life, reducing decay. The longest storage life was obtained using 800 μLL^−1^ of caraway oil (Aminifard and Bayat, 2018).

Thus, the evaluation of caraway oil's anti-pathogenic and antioxidant properties to maintain the quality of fruit and vegetable crops during the storage period is necessary. The present study aimed to evaluate the ability of two inexpensive natural substances, SA and caraway oil, to alleviate the postharvest CI in sweet pepper fruit. Hence, the pre-harvest foliar spraying of SA incorporated with postharvest caraway oil coating on CI, fruit quality, and antioxidant enzymes including SOD, POD, CAT, and capsaicin content in sweet pepper fruit was studied. *In vitro* antimicrobial assay of caraway oil on *B. cinerea* and decay incidence during storage period were also evaluated.

## Materials and methods

### Preparation of caraway oil

Seeds of Iranian caraway were purchased from a local market in Shahrood (36.4062° N, 55.0163° E). The caraway seeds were soaked in distilled water (2 h), and its oil was extracted using a Clevenger-type apparatus for steam distillation. Caraway powdered seed (500 g) and 2 L of water were placed into a 3-L round-bottom flask. The generated steam by heating the flask was condensed in a heat exchanger. Essential oil and aqueous phases were separated in the Clevenger head. Caraway oil was extracted for 4 h. Finally, it was dewatered, dried over anhydrous sodium sulfate, and stored at −20°C. To prepare caraway oil emulsion, 1.5, 0.3, and 0.6% (v/v) of caraway oil and Tween 80 as an emulsifier (10% of the volume of the caraway oil) were dissolved into distilled water (Rojas-Graü et al., 2007).

### Effect of caraway oil on the mycelium growth of fungus

The effect of caraway oil inhibition on radial mycelia growth was evaluated using the agar dilution method. Different aliquots of caraway oil were poured into 20 ml of autoclaved PDA culture medium (121°C for 15 min) to achieve the following concentrations: 0.15%, 0.3%, and 0.6%. The fungi disks (10 mm of 6-d-old culture) were placed at the center of Petri dishes. They incubated at 27°C for 7 d. The caraway oil-free medium was considered as control. The antifungal activity of caraway oil was determined as the inhibition of radial growth relative to the control at 7th d, based on the following formula: inhibition percentage = (RC – RT)/RC × 100, where RC and RT represent the average growth radius of the colonies from the control plate and the average growth radius of the colonies from the treatment plates, respectively (Achimón et al., 2021).

### Pepper fruit and experimental treatments

Sweet pepper seeds (*Capsicum annum* L. cv. Toronto) were planted in a commercial greenhouse in Fariman (35.7014° N, 59.8466° E), Razavi Khorasan, Iran. The nutrient and irrigation schedules were set up in accordance with a standard commercial greenhouse operation. Two concentrations of SA (1.5 and 3 mM) were sprayed twice at a 10-d interval before the harvesting stage, whereas the control plants received just distilled water as a spray. Sweet pepper fruit was harvested at commercial maturity stage (TSS = 6, L = 46.89, a = −5, b = 22). The harvested uniform-sized fruits were immediately transported to the horticultural laboratory and washed with 0.05% of sodium hypochlorite solution for 5 min. They were dried at room temperature and submerged in different concentrations of caraway oil (0.3 and 0.6%) and distilled water (control) for 5 min. Caraway oil (0.3 and 0.6%) (v/v) was mixed with Tween 80 (10% of the volume of the essential oil) to aid in distribution and complete incorporation. The distilled water was used to reach the final volume. Coated and control fruits were dried and stored for 60 d at 4 ± 2°C with 85–90% RH. Following 10, 20, 40, or 60 d of cold storage, fruits were moved to a controlled environment chamber and maintained at 20°C for 2 d (shelf life). In each treatment, four fruits (about 400 g) were used, and all experiments were repeated thrice. The physicochemical and biochemical experiments were performed at a 20-d interval. The composition of samples and their codes were: control (S0C0), S1C0 (1.5 mM SA foliar sprayed fruit), S2C0 (3 mM SA foliar sprayed fruit), S0C1 (0.3% caraway oil-coated fruit), S0C2 (0.6% caraway oil-coated fruit), S1C1 (1.5 mM SA foliar sprayed fruit + 0.3% caraway oil-coated fruit), S1C2 (1.5 mM SA foliar sprayed fruit + 0.6% caraway oil-coated fruit), S2C1 (3 mM SA foliar sprayed fruit + 0.3% caraway oil-coated fruit), and S2C2 (3 mM SA foliar sprayed fruit + 0.6% caraway oil-coated fruit).

### Chilling injury evaluation

CI incidence was evaluated according to the method described by Bar-Yosef et al. (2009). CI symptoms were investigated based on sunken pitting that it was more than 2 mm on the skin or calyx of sweet pepper fruits. CI was expressed as a percentage of damaged fruit from the total initial fruit number.

### Weight loss

Sweet pepper fruits were initially weighed at 0 and the end of each storage interval. The difference between the initial and final weight of fruit was considered weight loss based on following formula and was expressed as a percentage: Weight loss (%) = (w_1_-w_2_)/w_1_ × 100, where w_1_ is the initial weight, and w_2_ is the weight at the end of each storage interval.

### Firmness

Firmness was measured as puncture force in pepper fruit using a digital fruit hardness tester (model 41050, Germany) with a cylinder diameter of 2 mm. Firmness was measured at 3 points in the middle around the pepper fruit and stated as N.

### pH, total soluble solids (TSS), and titratable acidity (TA)

A homogeneous sample was prepared by blending the fruit in a blender. After mixing the sample, few drops were used to determine the pH and TSS. pH of the samples was measured by a pH meter (WTW model, Germany) according to AOAC (Chemists and Horwitz, 1975), and the TSS content of the fruit was determined using a handheld refractometer (ATAGO master 5 EM, Japan) and shown in Brix degree. TA was calculated according to the method of Mazumdar and Majumder (Mazumdar and Majumder, 2003) (with some modification) by titrating 2.5 ml of the fruit juice with 0.1 N NaOH and expressed as citric acid percentage.

### Color

Surface color changes of sweet pepper fruit were reported in L^*^ (higher positive values indicate more lightness, while lower values indicate darkness), a^*^, and b^*^ mode. It was measured at two opposite sides on the middle around of the fruit using Chroma Meter CR-400 (Konica Minolta Sensing, Japan). These values were then used to calculate chroma (C^*^= [a*^2^ + b*^2^]^1/2^), indicating the intensity or color saturation and the total color difference (ΔE^*^) = [ΔL^2^ + Δa^2^+ Δb^2^]^1/2^.

### MDA content and electrolyte leakage

MDA content of pepper fruit was measured according to Luo et al. (2012). Tissue samples (2 g) were frozen in liquid nitrogen, ground quickly, and extracted in 5 ml 10% (w/v) trichloroacetic acid (TCA). The homogenized sample was centrifuged at 10,000 × g for 15 min. In total, 2 ml of clear supernatant was mixed with 2 ml 10% (w/v) TCA containing 0.6% (w/v) thiobarbituric acid (TBA). The heating of the mixture was done at 100°C for 20 min. It was quickly cooled and centrifuged at 10,000 × g for 10 min. The absorbance of the supernatant at 450, 532, and 600 nm was measured by a Unico spectrophotometry (model 2150, China). The MDA content was calculated according to the formula: 6.25 × (OD_532_-OD_600_)-0.56 × OD_450_.

Electrolyte leakage was determined using a method described by Huang et al. (2012) with some modifications. Disks were taken with a cork borer (10 mm in diameter) from four fruits. A total of 12 disks were incubated in 15 ml of distilled water at 25°C (EC_1_) and shaken for 30 min. Another 12 disks were incubated in distilled water at 100°C for 25 min (EC_2_). The electrolyte leakage was determined with a conductivity meter (EC-400L). The relative electrolyte leakage was calculated according to the formula: Electrolyte leakage (%) = (EC_2_)/(EC_1_) × 100.

### Total phenolic content

To evaluate the total phenolic content, 4 g of frozen tissue samples were homogenized with 35 ml of cold methanol (70%). The homogenized sample was centrifuged at 13,000 × g at 4°C for 15 min. Supernatant (500 μL) was added to 1.5 ml of distilled water and 1 ml of Folin–Ciocalteu reagent at half strength; 1 ml of 10% (w/v) Na_2_CO_3_ was subsequently added and incubated at 25°C in darkness for 1.5 h. The absorbance was measured at 760 nm by a Unico spectrophotometry (model 2150, China). The standard curve of gallic acid was used to determine the total phenolic content, and the results were represented as mg gallic acid 100 g^−1^ FW, using a few modifications from the technique reported by Cong et al. (2017).

### Antioxidant enzyme activities

Frozen pulp weighting 2 g was homogenized in 10 ml of ice-cold extraction buffer containing 2 g of PVPP and ground at 4°C. The extraction buffer was 100 mM sodium phosphate (pH 7.8) for SOD and POD. For CAT, the extraction buffer was 100 mM sodium phosphate (pH 7.0). The homogenization was centrifuged at 15,000 × g for 30 min at 4°C, and the supernatant was used for the enzyme assay.

Catalase activity was assayed according to the method of Wang et al. (2005) with some modifications. In total, 1 ml of sodium phosphate buffer (50 mM, pH 7.0), 1 ml of 40 mM H_2_O_2_, and 1ml enzyme extract were used. A change of 0.01 per min in OD_240_ was considered one unit of CAT activity, and the results were expressed as U g^−1^ FW.

SOD activity was determined by the inhibition ability of SOD in the photochemical reduction of nitrotetrazolium blue chloride (NBT) in OD_560_ nm, according to the method proposed by Wang et al. (2005). The reaction mixture contained 75 μM NBT, 1-methionine 13 mM, EDTA. Na_2_ 10 μM, and 2 μM riboflavin in sodium buffer phosphate (0.05 M, pH 7.8). The reaction was started after adding riboflavin. The tubes containing the reaction mixture were placed under 60 μMm^−2^s^−1^ for 10 min, and the absorbance was recorded at 560 nm. The activity of SOD was expressed as U g ^−1^ FW.

For POD activity assay, 0.5 ml of enzyme extract was added into 2 ml sodium buffer phosphate 100 mM (pH 6.4) and 8 mM guaiacol for 30 min at 30°C. Then, 1 ml of H_2_O_2_ (24 mM) was added, and the increasing absorbance was recorded at 460 nm for 120 s (Wang et al., 2005). POD activity was expressed as U g ^−1^ FW.

### Decay incidence

Using the approach outlined by Zheng and Zhang (2002), the decay incidence was calculated. Based on a visual examination of the fruit's exterior, the incidence of decay was quantified. The following formula was used to calculate the decay incidence of sweet pepper fruits:


$$\begin{array}{l} \text{Decay }(\%)\text{ = number of fruit decayed/total number of fruits}\\ \;\times \text{ 100} \end{array}$$

### Capsaicin content

The capsaicin content of pepper fruit was performed according to the method of Sadasivam and Manickam (1996) with some modifications. Fruit pulp (3 g) was incubated in 150 ml ethyl acetate for 24 h. One ml of the extract was diluted to the volume of 5 ml using ethyl acetate. Then, 0.5 ml of vanadium oxychloride solution (vanadium oxychloride 0.5% in ethyl acetate) was added into the prepared mixture immediately before recording the absorbance of the samples. Pure capsaicin (0.01%) was prepared by adding 10 mg capsaicin in 100 ml of ethyl acetate and considered as standard. At 720 nm, the absorbance of the blank, standard, and sample was measured. The values were obtained before the vanadium oxychloride caused the color of the extract to change from green to yellow.

### Statistical analysis

The experiment was conducted as a completely randomized design (CRD) in a bi-factorial model (storage time × treatments) with triplicate, and standard deviation (SD) was calculated. The data were subjected to analysis of variance (ANOVA) within SAS statistical software (SAS 8.2 Institute Inc., Cary, NC, USA). Duncan's multiple range test (*p* < 0.05) was used to determine the difference.

## Results and discussion

### Effect of caraway essential oil on the mycelium growth of fungus

**Figure 1:**
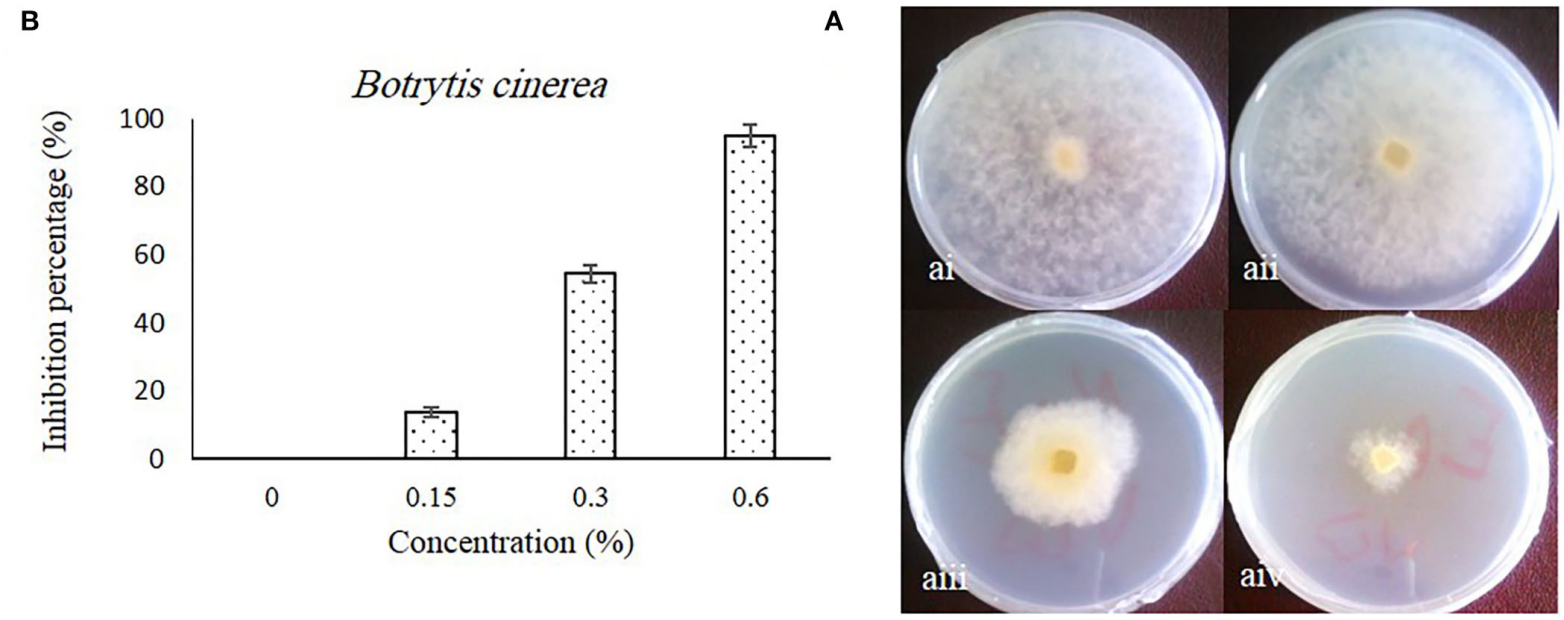
The effect of different concentrations of caraway oil on *B. cinerea* in *in vitro* after 7 d at 27°C. Colony morphology [**(A)** i, ii, iii, and iv]: **(A)** i, control; **(A)** ii, 0.15% of caraway oil; **(A)** iii, 0.3% of caraway oil; and **(A)** iv, 0.6% of caraway oil. The inhibition percentage of caraway oil on *B. cinerea* **(B)**. Values represent mean ± SD of three replications. Different letters indicate significant differences at the 5% level according to the Duncan test.

The inhibitory effect of caraway oil on *B. cinerea* is shown in Figure 1. The obtained results showed that the growth zone of *B. cinerea* mycelia on PDA medium supplemented with caraway oil was significantly inhibited by increasing the caraway oil concentration compared to the caraway oil-free medium (Figure 1A). The highest inhibition percentage of mycelium growth was observed in the medium with 0.6% of caraway oil, while the inhibition percentage mycelium growth in the medium with 0.3 and 0.15% of caraway oil was obtained as 54.43 and 13.9%, respectively (Figure 1B). The principal components of caraway oil, such as carvone, limonene, β-selinene, belemene, and caryophyllene oxide, belong to terpenes. The antifungal activity of these components has been previously reported (Gniewosz et al., 2013; Seidler-Łozykowska et al., 2013). Their antifungal activity is related to lipophilic property that gives them the ability to penetrate cell walls. Then, their reaction with the cytoplasm in the hyphae results in the death of the mycelium (Sharma and Tripathi, 2006). Therefore, the ability of caraway oil as an edible coating at 0.3 and 0.6% concentrations on postharvest storage of sweet pepper fruits was investigated.

### Chilling injury (CI)

**Figure 2:**
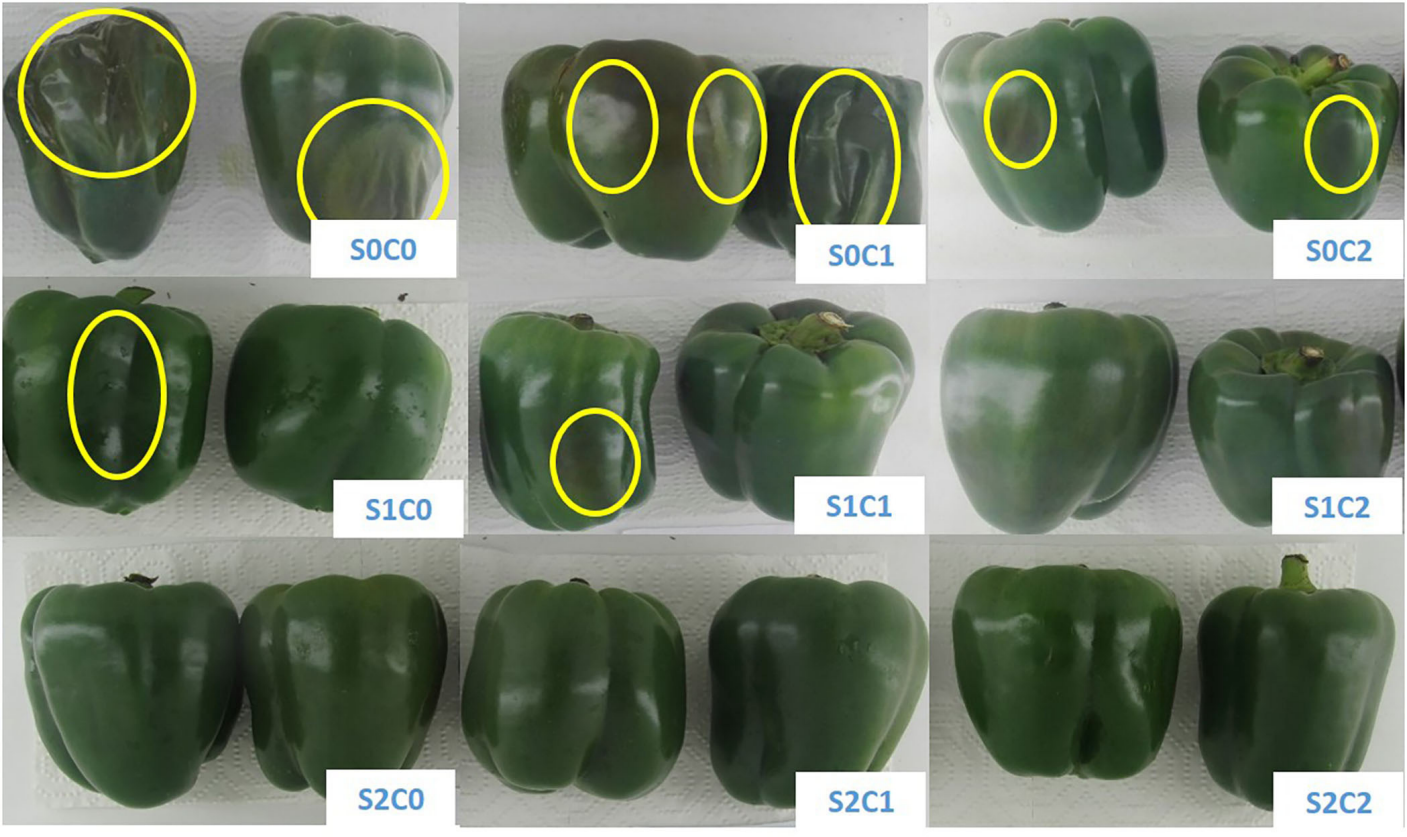
Sweet pepper fruit after 60 d of storage period at 4 ± 2°C plus shelf life (2 d, 20°C).

**Figure 3:**
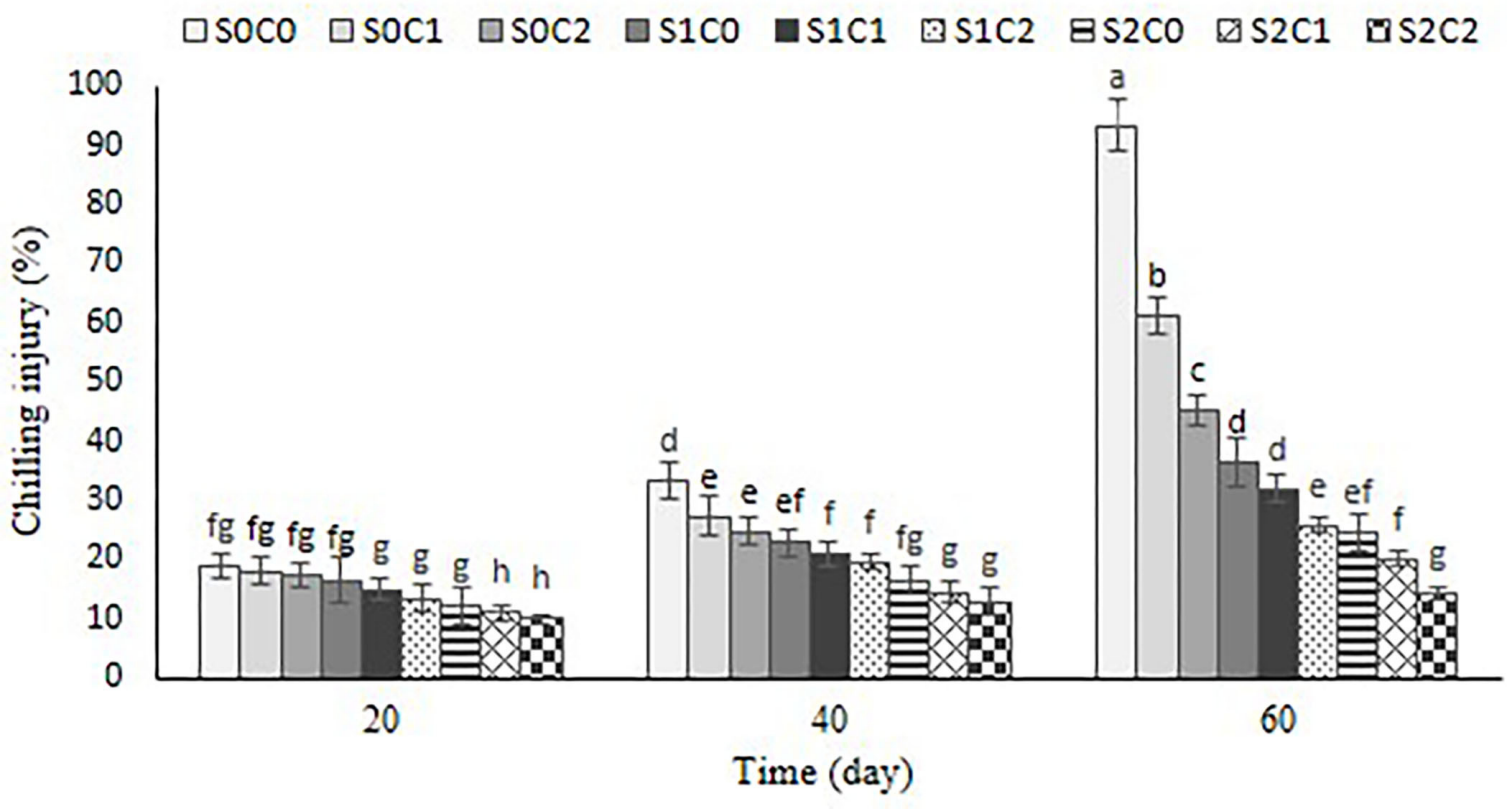
Effect of foliar spraying of SA and caraway oil coating on chilling injury percentage during 60 d of storage at 4 ± 2°C plus shelf life (2 d, 20°C). S0C0: control, S0C_1_: 0.3% caraway oil, S0C_2_: 0.6% caraway oil, S1C0: 1.5 mM Salicylic acid, S1C1: 1.5 mM Salicylic acid + 0.3% caraway oil, S1C2: 1.5 mM Salicylic acid + 0.6% caraway oil, S2C0: 3 mM Salicylic acid, S2C1: 3 mM Salicylic acid + 0.3% caraway oil, S2C2: 3 mM Salicylic acid + 0.6% caraway oil. Values represent mean ± S.D of three replications. Different letters indicate significant differences at the 5% level according to the Duncan test.

The first CI symptoms were observed as the sunken lesions and pitting on the fruit surface of control and C1 treatments after 20th d, but these symptoms were observed in smaller sizes after 40 d in rest treatments. The CI index of the control and C1 treatments advanced quickly over the course of 60 d, as illustrated in Figure 2. By increasing SA and caraway oil treatment concentrations both alone and when combined, CI symptoms were reduced. The treatment of C2S2 dramatically reduced the increased CI index, having a value of about 14.36% compared with control (93.33%) at the end of cold storage (Figure 3).

Inconsistent with the previous findings in SA application on peach (Yang et al., 2012), cucumber (Cao et al., 2009), sponge ground (Cong et al., 2017), and cinnamon oil on shitake mushroom (Jiang et al., 2015), incorporation of SA and caraway oil significantly alleviated CI in sweet pepper fruit during the extend of the experiment until 60 d. One of the main causes of CI is the overproduction of active oxygen species (Lurie et al., 1987; Stanley and Parkin, 1991). SA, a critical signaling molecule, is crucial to the defensive mechanisms used by plants to combat freezing stress. In a research, Buchanan et al. (2015) showed the favorable impact of SA on the inhibition of postharvest oxidative stress, which is consistent with our findings. They stated that SA alleviates CI *via* an increase in ROS avoidance genes, including alternative oxidase (AOX) and ROS scavenging genes. Moreover, the increase in AOX transcript levels and the reduction of CI symptoms using SA and MeSA were reported in green bell peppers (Fung et al., 2004). A previous study has reported that essential oil of clove, cinnamaldehyde, and thyme can reduce the increase of H_2_O_2_ content and O_2_- production rate during storage (Jiang et al., 2015).

### Weight loss

**Figure 4:**
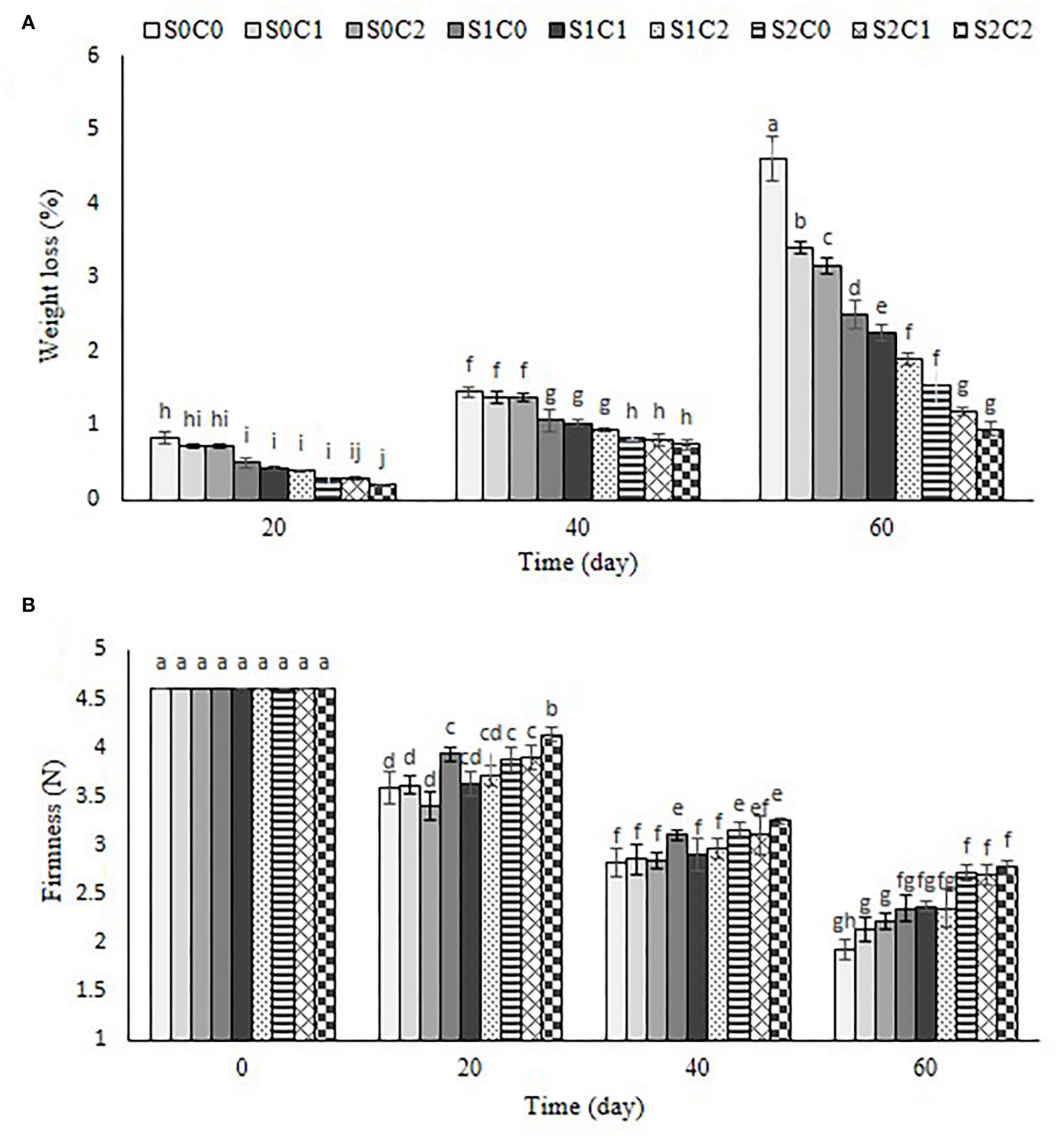
Effect of foliar spraying of SA and caraway oil coating on weight loss **(A)** and firmness **(B)** during 60 d of storage at 4 ± 2°C plus shelf life (2 d, 20°C). S0C0: control, S0C1: 0.3% caraway oil, S0C2: 0.6% caraway oil, S1C0: 1.5 mM Salicylic acid, S1C1: 1.5 mM Salicylic acid + 0.3% caraway oil, S1C2: 1.5 mM Salicylic acid + 0.6% caraway oil, S2C0: 3 mM Salicylic acid, S2C1: 3 mM Salicylic acid + 0.3% caraway oil, S2C2: 3 mM Salicylic acid + 0.6% caraway oil. Values represent mean ± S.D of three replications. Different letters indicate significant differences at the 5% level according to the Duncan test.

The weight loss increased by passing the storage period in the control, and all treated fruit and it was significantly affected by SA and caraway oil treatments (Figure 4A). The maximum physiological weight loss was recorded in control fruits so that they lost 4.6% of their initial weight by the end of the storage period. Throughout the storage period, 3 mM of SA foliar spraying incorporated with 6% of caraway oil coating (S2C2) (0.95%) resulted in the least weight loss. The treated fruit with 3 mM of SA (S2C0) maintained lower weight loss than the treated fruit with caraway oil alone. Weight loss is one of the principal physiological factors which negatively affects pepper quality during storage period and subsequent marketing (Smith et al., 2006). Gradual increase in weight loss throughout the cold storage, regardless of treatment, could be related to water loss resulting from transpiration and respiration processes in the fruit (Abbasi et al., 2011). To increase the amount of time peppers may be stored, appropriate techniques must be used to reduce the rate of water loss. SA as an electron donor creates free radicals that can interfere with normal respiration. It can diminish respiration rate and transpiration velocity by shutting off stomata (Shafiee et al., 2010). Reduced water loss in caraway oil-coated sweet pepper could be due to the antimicrobial properties of caraway oil against spoilage pathogens and preventing cell wall degradation, which leads to the preservation of the cell water content, the reduction of respiration intensity, and rate of metabolic reaction.

Besides, reduced weight loss in caraway oil-coated fruit could be in terms of the blockage of stomata and guard cells that ultimately slowed down the active metabolic processes and respiration. Reduced weight loss in caraway oil-treated fruit could be attributed to the semipermeable effect of caraway oil coatings during moisture loss, respiration, and movements of solutes across the membrane. A similar reduction in fruit weight loss has been reported in bell pepper when coated with different concentrations of cinnamon oil (Ullah et al., 2017).

### Firmness

The control fruit's firmness dramatically decreased over the course of 60 d of storage, but the addition of either SA foliar spraying or caraway oil coating, alone or in combination, greatly improved fruit firmness throughout the course of the whole storage period (Figure 4B). Moreover, foliar sprayed peppers with 3 mM SA showed a higher firmness than caraway oil-coated pepper in both concentrations (S0C1 and S0C2). The most increased firmness was noticed as 2.76, 2.71, and 2.68 N in S2C1, S2C0, and S2C2 treatments, respectively.

A gradual decrease in fruit firmness during the storage period reflects the water content and metabolic changes. Enhanced firmness, a critical indicator of delayed ripening, was obvious in SA foliar spraying, caraway oil coating individually, and their incorporation compared to control fruits during the storage period. The retention of fruit firmness as the result of SA treatment was shown that SA decreases ethylene production and inhibits cell wall and membrane degradation by slowing down the activity of degrading enzymes such as polygalacturonase (PG), lipoxygenase (LOX), cellulose, and pectin methylesterase (PME) leading to decreasing the fruit softening rate (Srivastava and Dwivedi, 2000; Zhang et al., 2003). In the previous study, cell walls were altered by the activation of softening enzymes, including polygalacturonase and pectinesterase, and resulted to softening in nectarine fruits (Manganaris et al., 2005). According to Shehata et al., citrus essential oil coatings hindered the pectin enzyme by delaying the metabolic processes and keeping strawberry fruit firmness (Shehata et al., 2020). Our findings are similar to those reported in previous studies from kiwifruit subjected to postharvest SA (Zhang et al., 2003) and in bell pepper fruit coated with cinnamon oil (Ullah et al., 2017). The mentioned treatments reduced the softness of kiwifruit and bell pepper fruits and were suggested to delay ripening and senescence during their storage.

### Total soluble solids (TSS), titratable acidity (TA), and pH

A gradual increase in TSS was observed in control and all treated fruit during the 60 d of storage period. A lower TSS was exhibited in S2C1 and S2C2 treatments until 60th d (*p*< *0.05*) compared with control (8.50 and 9.66, respectively), whereas TSS of S1C0, S2C0, S0C2, S1C1, and S1C2 treatments ranged between 8.76 and 8.9. S0C1 treatment and control showed no significant difference (Table 1).

**Table 1:** Effect of pre-harvest foliar spray of SA and caraway oil-coating on pH, titrable acidity (TA) and total soluble solids (TSS) of pepper fruits during cold storage period.

**Storage time (days)**	**Treatments**								
	**Control (S0C0)**	**S1C0**	**S2C0**	**S0C1**	**S0C2**	**S1C1**	**S1C2**	**S2C1**	**S2C2**
***pH***									
0	5.15^j^	5.15^j^	5.15^j^	5.15^j^	5.15^j^	5.15^j^	5.15^j^	5.15^j^	5.15^j^
20	5.73 ± 0.15^hi^	5.56 ± 0.09^ij^	5.94 ± 0.11^g−i^	5.38 ± 0.03^jk^	5.31 ± 0.05^jk^	5.77 ± 0.17^hi^	5.48 ± 0.05^jk^	5.68 ± 0.16^hi^	5.24 ± 0.16^k^
40	6.20 ± 0.07^e−f^	6.36 ± 0.06^d−f^	6.42 ± 0.25^c−e^	6.15 ± 0.11^fg^	6.24 ± 0.09^e−f^	6.26 ± 0.18^e−f^	6.20 ± 0.45^fg^	6.24 ± 0.09^ef^	6.06 ± 0.06^gh^
60	6.83 ± 0.012^a^	6.58 ± 0.09^a−d^	6.55 ± 0.52^a−d^	6.77 ± 0.17^ab^	6.60 ± 0.18^a−d^	6.57 ± 0.08^a−d^	6.50 ± 0.12^b−e^	6.47 ± 0.07^b−e^	6.39 ± 0.07^c−f^
***TA***									
0	31.04^a^	31.04^a^	31.04^a^	31.04^a^	31.04^a^	31.04^a^	31.04^a^	31.04^a^	31.04^a^
20	28.05 ± 0.83^ab^	28.30 ± 0.50^ab^	23.72 ± 0.55^d^	26.92 ± 0.64^bc^	29.71 ± 0.23^a^	26.53 ± 0.32^bc^	27.09 ± 0.61^bc^	25.23 ± 1.34^cd^	28.20 ± 0.92^ab^
40	13.36 ± 2.01^e^	16.25 ± 1.00^d^	15.03 ± 0.70^d^	15.82 ± 0.96^d^	15.95 ± 0.88^d^	15.11 ± 0.64^d^	16.59 ± 0.25^d^	15.36 ± 0.72^cd^	18.04 ± 0.65^c^
60	7.27 ± 0.83^i^	8.29 ± 0.98^h^	8.66 ± 1.28^gh^	8.14 ± 0.97^h^	8.64 ± 1.16^gh^	8.81 ± 1.18^g^	8.99 ± 2.02^g^	10.56 ± 1.09^f^	10.70 ± 1.12^f^
***TSS***									
0	6.00^m^	6.00^m^	6.00^m^	6.00^m^	6.00^m^	6.00^m^	6.00^m^	6.00^m^	6.00^m^
20	7.33 ± 0^i^	7.66 ± 0.5^gh^	7.24 ± 0^ij^	7.16 ± 0^ij^	7.16 ± 0.28^ij^	7.16 ± 0.28^ij^	7.13 ± 0.28^ij^	7.16 ± 0.28^ij^	7.00 ± 0.28^k^
40	8.66 ± 0^d^	8.00 ± 0.28 ^fg^	7.66 ± 0.57^gh^	8.00 ± 0.28^fg^	8.00 ± 0.28^fg^	7.83 ± 0.28^g^	7.73 ± 0.5^gh^	7.66 ± 0^gh^	8.00 ± 0.5^fg^
60	9.66 ± 0.28^a^	8.76 ± 0^bcd^	8.80 ± 0.28^bcd^	9.16 ± 0.28^ab^	8.81 ± 0.28^bcd^	8.90 ± 0.5^bcd^	8.83 ± 0.28^bcd^	8.50 ± 0^ef^	8.50 ± 0.5^ef^

Means with the same letters within row are not significantly different at p < 0.05 using Duncan. Control (S0C0), S_1_C0 (1.5 mM SA foliar sprayed fruit), S2C0 (3 mM SA foliar sprayed fruit), C1S0 (0.3 % caraway oil coated fruit), C2S0 (0.6 % caraway oil coated fruit), S1C1 (1.5 mM SA foliar sprayed fruit + 0.3 % caraway oil coated fruit), S1C2 (1.5 mM SA foliar sprayed fruit + 0.6 % caraway oil coated fruit), S2C1 (3 mM SA foliar sprayed fruit + 0.3 % caraway oil coated fruit), and S2C2 (3 mM SA foliar sprayed fruit + 0.6 % caraway oil coated fruit).

A similar effect was reported by Ullah et al. (2017) in bell pepper coated with cinnamon oil and Rao et al. (2011) in sweet pepper postharvest treated with SA, respectively.

Considering TA, increased concentrations of SA and caraway oil were effective in retaining the TA content of pepper fruit during storage. The findings demonstrated that TA reduced with increasing storage time for both treated and untreated fruit. Using SA and caraway oil resulted in higher TA than control. Among all treatments, S2C1 and S2C2 showed a lower decrease in TA content while a higher reduction in TA was observed in control (Table 1).

The pH of sweet pepper fruit was increased during the storage period. The gradual increase in pH with the passage of the storage period was observed (Table 1). Foliar spraying of SA incorporated with caraway oil and their individual applications at higher concentration significantly affected pH changes of sweet pepper fruit during storage. At the 60th d of storage, a lower pH was observed in S2C1 and S2C2 (6.39 and 6.47, respectively), followed by S1C2 (6.50) treatments. The higher pH value was observed in S0C1 treatment and control (6.77 and 6.88, respectively).

Our results showed that SA treatment lowers the TSS value, which represented that SA delays the fruit softening process. Similar results were reported by Rao et al. (2011) in sweet pepper fruit treated with SA at the postharvest stage. They stated that the slowing down of respiration rate and metabolic activity, which results in a slowdown in the ripening process, may be the cause of the maintenance of TSS in treated fruit. The caraway oil coating results are in accordance with previous findings (Ullah et al., 2017). The authors reported a lower increase in TSS of coated bell pepper fruit with cinnamon oil because using these oils as a protecting barrier can delay the ripening process in treated fruit. The preservation of TA content was directly proportional to SA and caraway oil concentrations. The retention of acidity in treated fruit was probably in terms of decreasing the metabolic changes of organic acid into carbon dioxide and water. A lower decrease in TA of mandarin and bell pepper when these fruits were treated with SA and cinnamon oil during the postharvest period was reported, previously (Ullah et al., 2017; Haider et al., 2020).

A tendency to increase the pH values (*p* < 0.05) was observed in control and all treated fruit with the passage of time. The pH of the sweet pepper fruit was preserved during cold storage as a result of the use of SA and caraway oil treatments. This tendency could relate to reducing the organic acid content of the stored fruits (Benítez, 2013). Similarly, Rao et al. (2011) and Treviño-Garza et al. (2017) have reported the positive effect of SA and edible coating to maintain pH in sweet pepper and fresh-cut pineapple, respectively.

### Color

**Figure 5:**
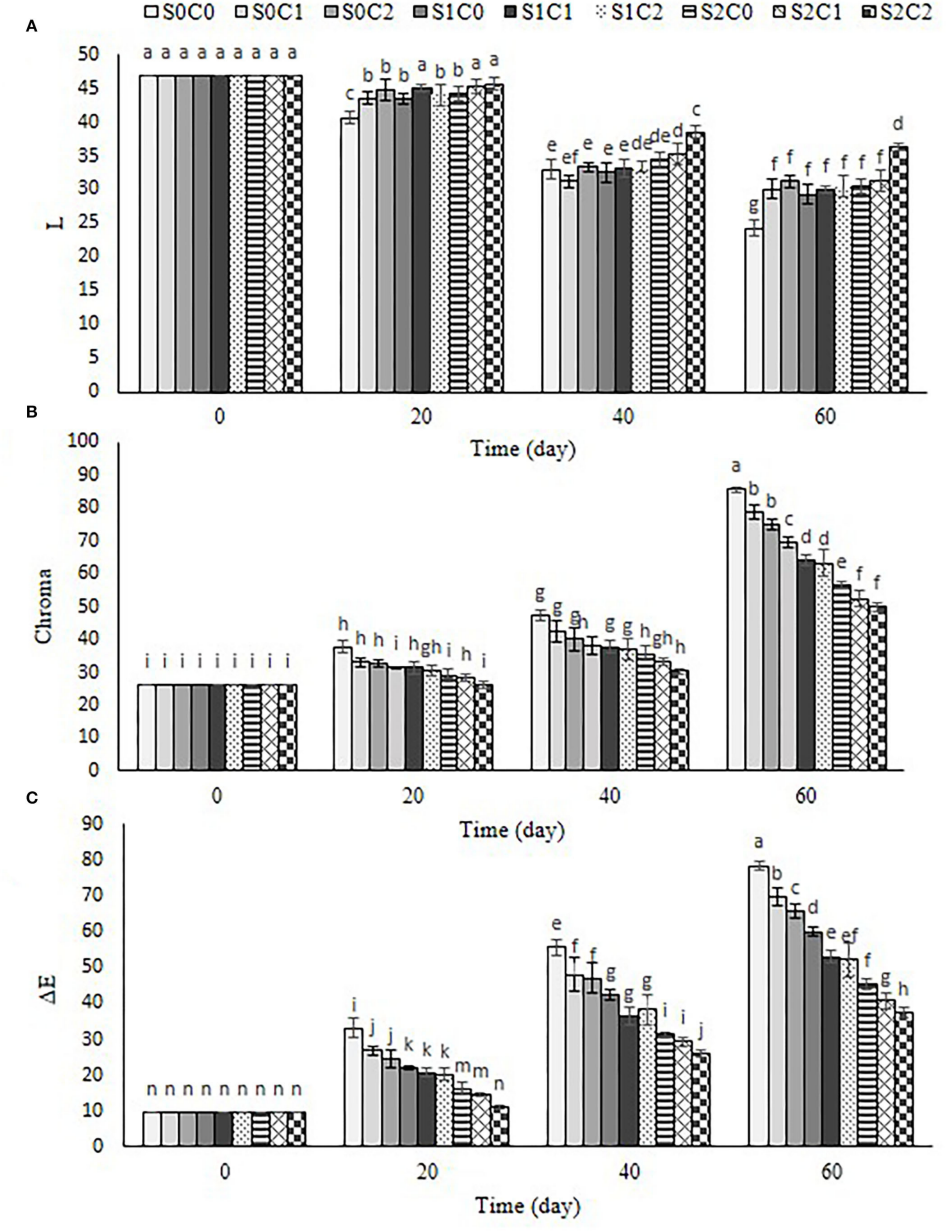
Effect of foliar spraying of SA and caraway oil coating on L*(A), chroma **(B)**, and ΔE **(C)** during 60 d of storage at 4 ± 2°C plus shelf life (2 d, 20°C). S0C0: control, S0C1: 0.3% caraway oil, S0C2: 0.6% caraway oil, S1C0: 1.5 mM Salicylic acid, S1C1: 1.5 mM Salicylic acid + 0.3% caraway oil, S1C2: 1.5 mM Salicylic acid + 0.6% caraway oil, S2C0: 3 mM Salicylic acid, S2C1: 3 mM Salicylic acid + 0.3% caraway oil, S2C2: 3 mM Salicylic acid + 0.6% caraway oil. Values represent mean ± S.D of three replications. Different letters indicate significant differences at the 5% level according to the Duncan test.

Evaluation changes in the color of skin by monitoring lightness (L^*^), chroma, and total color change (ΔE^*^) are represented in Figure 5. The results showed that the lightness (L^*^) of sweet pepper fruit decreased (Figure 5A) chroma, and ΔE^*^ increased with the progression of the storage period in control and all treated fruit (Figures 5B,C). The lightness of external color of fruit decreased gradually by 20th d after that intensively decreased by 60th d, while a reverse trend was observed for chroma and ΔE^*^ in control and all treated fruit. The lightness of external color of treated fruit with higher concentration of SA foliar spraying incorporated with caraway oil coating (S2C2) indicated a 1.49-fold higher than the control fruit. The maximum value of chroma and ΔE^*^ was equal to 85.64 and 78.42 in the control, while the minimum of these values was observed as 49.84 and 37.48 and in S2C2 treatment (Figure 5C).

The values of chroma and ΔE^*^ were significantly lower in S2C2 treatment (1.7- and 2.09-fold less) as compared to the control fruit. The skin color of the fruit is considered a critical parameter in determining sweet pepper quality during postharvest storage. It appears that the preservation of color parameters in sweet pepper fruits was positively impacted by SA foliar spraying, caraway oil coating, and their combination. The color change of green pepper is related to the exchange of chloroplast to chromoplast where conversion in pigment content of sweet pepper increases as senescence advances (Gong and Mattheis, 2003). Moreover, the effect of SA on a delay color change in sweet pepper fruit is associated with delay in chlorophyll degradation and carotenoid production. An increase of chroma and ΔE^*^ could be in terms of the increase in a^*^ and/or b^*^ value showing the loss of green color and synthesis of lycopene and β-carotene along with senescence progression. An ability of caraway oil coating to delay color change in sweet pepper fruit may be related to respiration reduction and the aging process, as Gao et al. (2014) and Ullah et al. (2017) have reported.

### MDA content and electrolyte leakage

**Figure 6:**
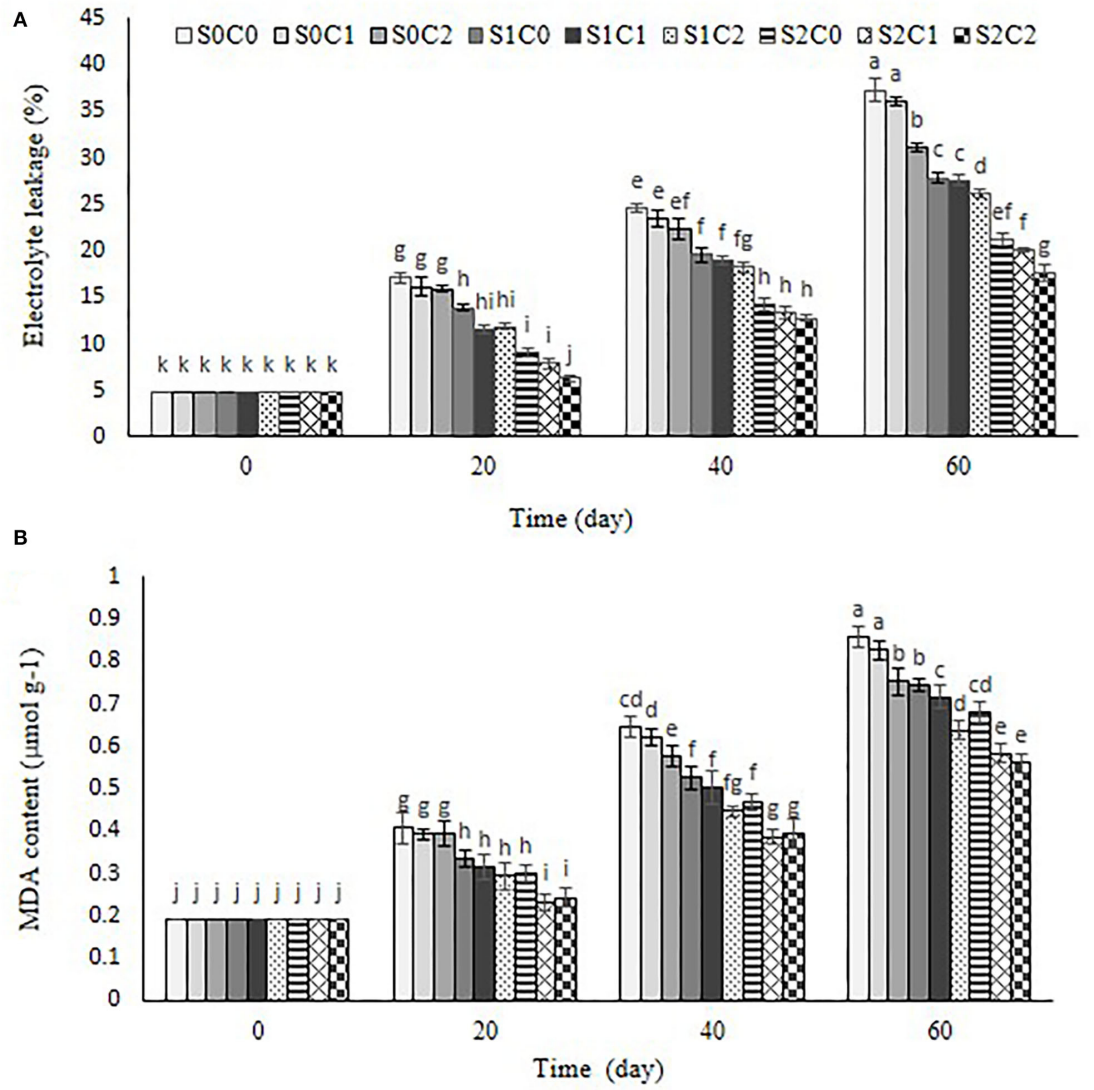
Effect of foliar spraying of SA and caraway oil coating on electrolyte leakage **(A)** and MDA **(B)** during 60 d of storage at 4 ± 2°C plus shelf life (2 d, 20°C). S0C0: control, S0C1: 0.3% caraway oil, S0C2: 0.6% caraway oil, S1C0: 1.5 mM Salicylic acid, S1C1: 1.5 mM Salicylic acid + 0.3% caraway oil, S1C2: 1.5 mM Salicylic acid + 0.6% caraway oil, S2C0: 3 mM Salicylic acid, S2C1: 3 mM Salicylic acid + 0.3% caraway oil, S2C2: 3 mM Salicylic acid + 0.6% caraway oil. Values represent mean ± SD of three replications. Different letters indicate significant differences at the 5% level according to the Duncan test.

As shown in Figure 6A, electrolyte leakage and MDA showed a similar trend (Figure 6B), so these increased in control and all treated fruit during the storage period. The highest level of electrolyte leakage was observed in the control fruit (37.3%) and S0C1 treatment (36.06%) (Figure 6A). At the end of storage, electrolyte leakage in S2C2 (17.66%), S2C1 (20.03%), and S2C0 (21.2%) treatments was lower than in other treatments (Figure 6A).

MDA content continuously increased in control and all treated fruit during storage at 4 ± 2°C. MDA content of sweet pepper fruit was treated with higher concentration of SA foliar spraying (3 mM) and was diminished more than the treatments of caraway oil alone. The incorporation of SA foliar spraying with caraway oil coating showed a significant effect on the inhibition of the membrane injury; hence, the lowest MDA content was observed in S2C1 (0.58 μmol g^−1^) and S2C2 (0.56 0.58 μmol g^−1^) treatments compared to control fruit (0.85 μmol g^−1^). Results showed that membrane integrity maintained better in the treated fruit due to inhibited accumulation of MDA content and electrolyte leakage reduction (Figure 6B).

The main effect of CI is to alter the fatty acid phospholipid composition at the cell membrane, which is followed by a series of secondary reactions that disturb the structure of the cell (Lurie et al., 1987; Stanley and Parkin, 1991). The suitable indications for evaluating the effects of oxidative stress damage are MDA and electrolyte leakage (Luo et al., 2015). Membrane damage and deterioration rate are commonly related to increasing electrolyte leakage (Kwon et al., 2002; Yabuta et al., 2002; Xu et al., 2019) A lipid peroxidation resulting from ROS accumulation leads to membrane damage and increases electrolyte leakage and MDA content (Repetto et al., 2012). Low accumulation of MDA content and stability of the membrane in the plant tissue represent the integrity of bio-membranes subjected to low temperature (Endo et al., 2019). The results of the present study approved that the incorporation of SA foliar spraying and caraway oil coating significantly inhibited the increasing MDA content and electrolyte leakage in sweet pepper fruit stored at low temperature. Therefore, maintenance of membrane integrity is important in the resistance to chilling stress during cold storage. Similar results were achieved to inhibit MDA accumulation and electrolyte leakage in immersed sponge ground in SA solution (Cong et al., 2017) and sweet pepper coated with cinnamon oil (Xing et al., 2011).

### Total phenolic content

**Figure 7:**
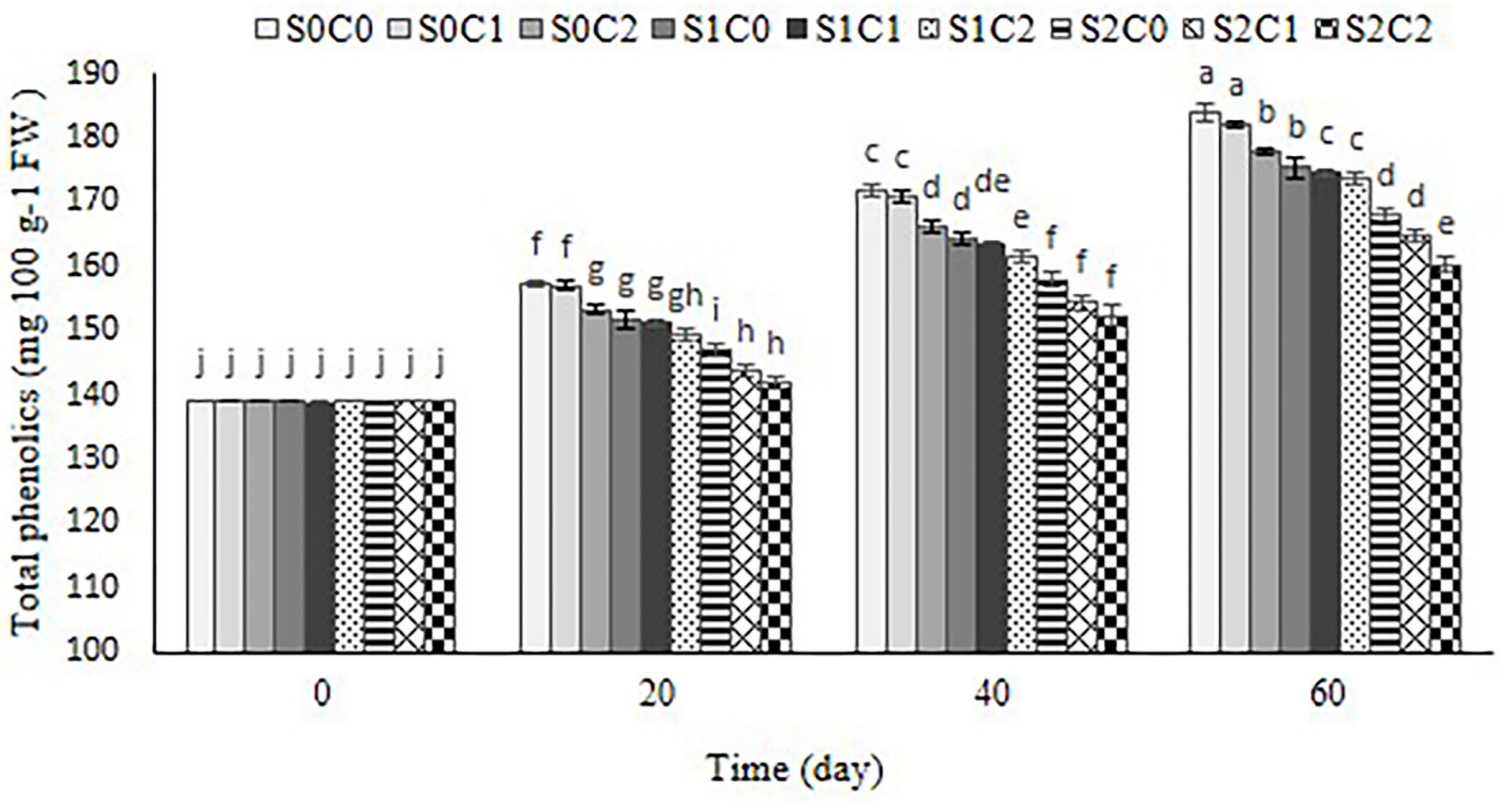
Effect of foliar spraying of SA and caraway oil coating on total phenolic during 60 d of storage at 4 ± 2°C plus shelf life (2 d, 20°C). S0C0: control, S0C1: 0.3% caraway oil, S0C2: 0.6% caraway oil, S1C0: 1.5 mM Salicylic acid, S1C1: 1.5 mM Salicylic acid + 0.3% caraway oil, S1C2: 1.5 mM Salicylic acid + 0.6% caraway oil, S2C0: 3 mM Salicylic acid, S2C1: 3 mM Salicylic acid + 0.3% caraway oil, S2C2: 3 mM Salicylic acid + 0.6% caraway oil. Values represent mean ± SD of three replications. Different letters indicate significant differences at the 5% level according to the Duncan test.

As shown in Figure 7, total phenolic content increased with the passage of the storage period in control and all treated fruit. SA foliar spraying incorporated with caraway oil coating significantly restrained the increase of total phenolic content in sweet pepper fruit. Thus, total phenolic content in SA (S2C0) and SA incorporated with caraway oil treatments (S2C2) were 9% and 13% lower than control fruit, respectively (*p*< *0.05*) (Figure 7).

Our observation indicated that the increase of total phenolic content resulting from CI could be alleviated by SA and caraway oil treatments. This result was in agreement with the previous study of the bamboo shoot (Luo et al., 2012) and sponge ground (Cong et al., 2017) that the alleviation of CI by SA was associated with decreased total phenolic content and PAL and PPO activity. One of the typical reactions of plants to biotic and abiotic stresses is the accumulation of secondary metabolites like phenolic (Dong et al., 2012). The essential oil would function as a signaling substance that triggers a signal resembling moderate stress. In response to this stress, additional phenolic compounds are produced in the fruit (Sharma and Tripathi, 2006).

### Antioxidant enzymes

**Figure 8:**
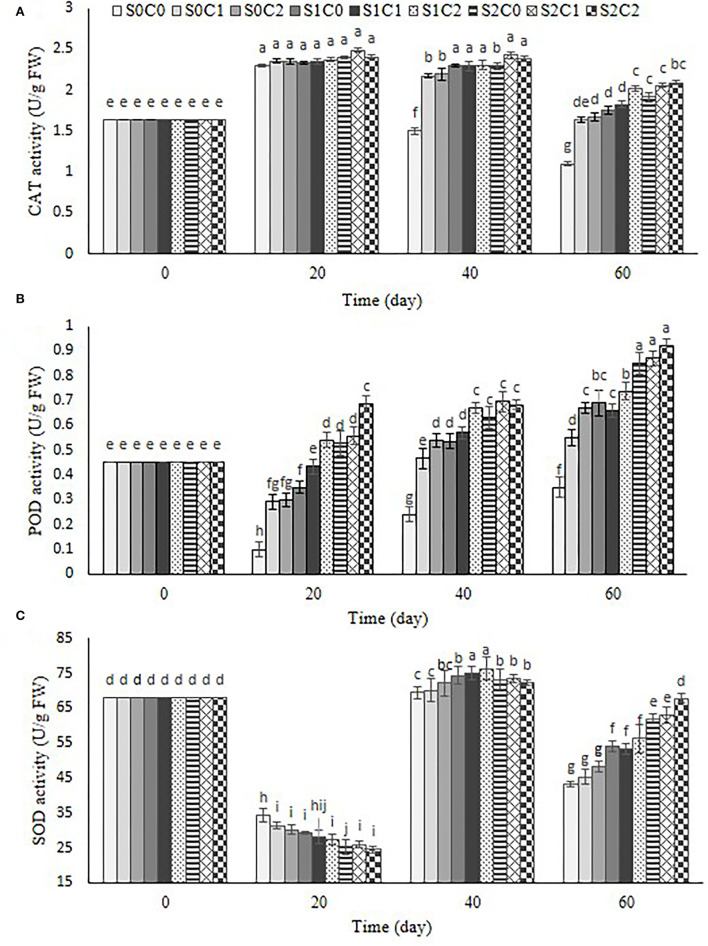
Effect of foliar spraying of SA and caraway oil coating on CAT **(A)**, POD **(B)**, and SOD **(C)** during 60 d of storage at 4 ± 2°C plus shelf life (2 d, 20°C). S0C0: control, S0C1: 0.3% caraway oil, S0C2: 0.6% caraway oil, S1C0: 1.5 mM Salicylic acid, S1C1: 1.5 mM Salicylic acid + 0.3% caraway oil, S1C2: 1.5 mM Salicylic acid + 0.6% caraway oil, S2C0: 3 mM Salicylic acid, S2C1: 3 mM Salicylic acid + 0.3% caraway oil, S2C2: 3 mM Salicylic acid + 0.6% caraway oil. Values represent mean ± SD of three replications. Different letters indicate significant differences at the 5% level according to the Duncan test.

Our observation revealed that the CAT activity increased in control and treated fruit up to 20th d and 40th d, respectively, and then reduced. At the end of the storage period, the highest CAT activity was obtained in S1C2 (0.73 U g^−1^ protein), S2C1 (0.87 U g^−1^ protein), and S2C2 (0.92 U g^−1^ protein) treatments during storage period, but the minimum was obtained in control (Figure 8A).

As can be observed in Figure 8B, a decrease in POD activity was observed during 20 d of the storage period in control fruit, S0C1, S0C2, and S1C0 treatments, after that increased until the end of the storage period, while a steady increase for POD activity was found in S2C0 and SA foliar spraying incorporated with caraway oil coating (S1C1, S1C2, S2C1, and S2C2) up to 60th d of the storage period; hence, the highest POD activity was observed as 0.92 U g^−1^ protein in S2C2 treatment, while the lowest enzyme activity was obtained as 0.35 U g^−1^ protein in control fruit.

By the progression of the storage period, a decrease in SOD activity was initially observed in control and all treated fruit. Up to the 40th d, this enzyme activity grew and then it declined until the conclusion of the storage period (Figure 8C). After the storage time, it was discovered that SA foliar spraying combined with caraway oil had a greater activity than control fruit and separate applications of SA and caraway oil treatments. However, the decrease in antioxidant activity after a specific time of storage could be resulted from their sensitivity to the low temperature (Haider et al., 2020). The maximum and minimum SOD activity were observed as 67.8 U g^−1^ protein and 43.3 U g^−1^ protein in S2C2 treatment and the control fruit, respectively (Figure 8C).

CI is considered to be associated with the defensive system, including CAT, SOD, and POD antioxidant enzymes (Xu et al., 2009; Cong et al., 2017). SOD, as a major O_2_- scavenging enzyme, catalyzes O_2_- radicals into H_2_O_2_ and O_2_, while the degradation of H_2_O_2_ to H_2_O and O_2_ can be done by CAT and POD enzymes. The mechanism of SA in alleviating CI could be attributed to enhance the membrane integrity and antioxidant system activity (Aghdam and Bodbodak, 2013). Based on the previous studies on cucumber (Cao et al., 2009), peach (Yang et al., 2012), and mandarin (Haider et al., 2020), postharvest SA treatment maintained higher activities of CAT, POD, and SOD in treated fruit compared with control fruit. It was found that SA, as one of the most important phenolic compounds, can induce the expression of ROS genes, including alternative oxidase (AOX) (Asghari and Aghdam, 2010), and fortify the fruit defense system by stimulating the synthesis of antioxidant enzymes (Huang et al., 2008). In the pre-harvest SA treatments in sweet cherry and peach, there was an increase in the antioxidant capacity compared with the control (Giménez et al., 2014; Erogul and Özsoydan, 2020). Our findings showed that incorporation of SA foliar spraying and caraway oil coating resulted in the highest antioxidant enzyme activity and the lowest CI in treated pepper fruit. The activity of antioxidant enzymes including CAT, POD, and SOD is a part of the defense system in plants against various abiotic and biotic stresses, and they help to scavenge free radicals which damage the vital cell membrane under stress conditions (Sels et al., 2008).

### Decay incidence

**Figure 9:**
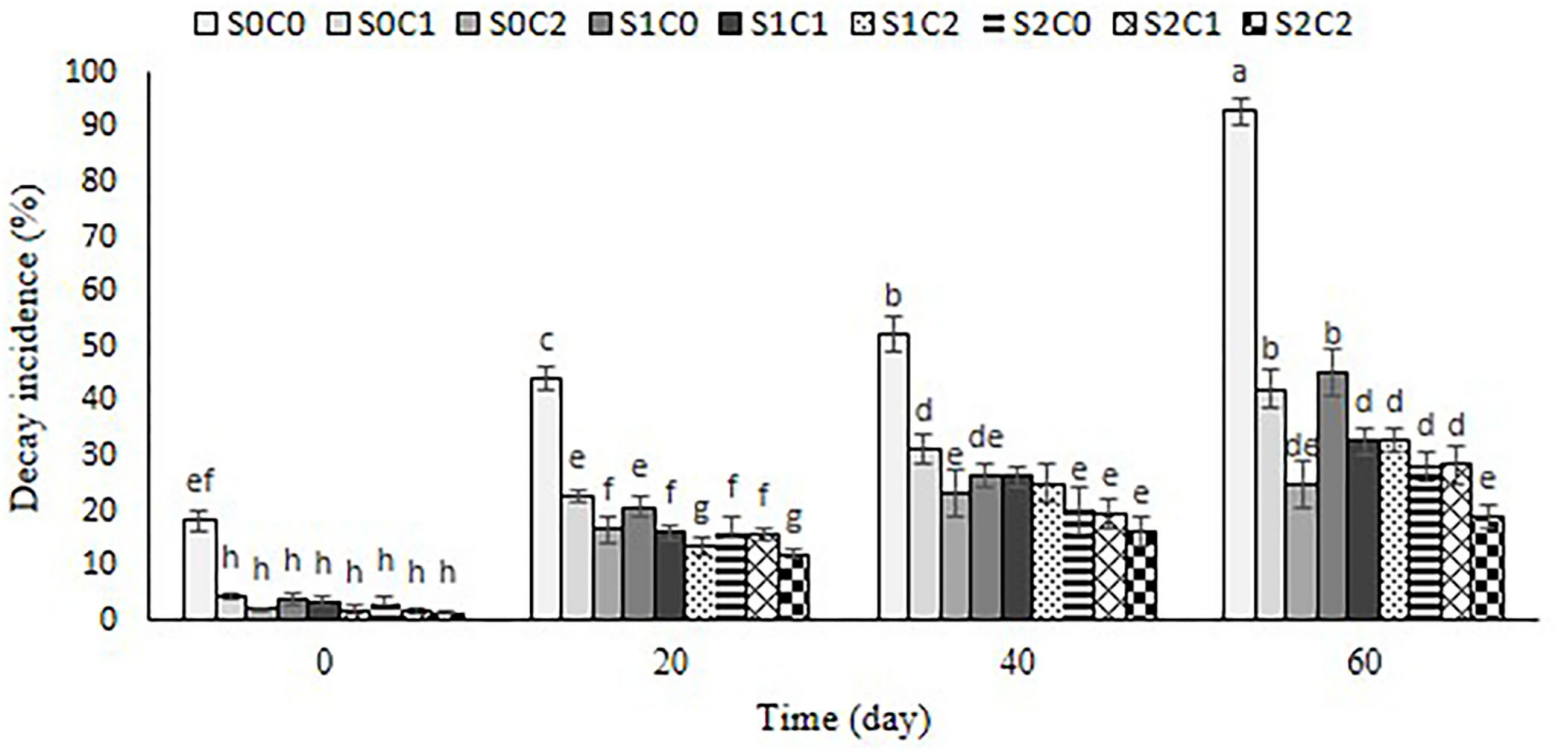
Effect of foliar spraying of SA and caraway oil coating on decay incidence during 60 d of storage at 4 ± 2°C plus shelf life (2 d, 20°C). S0C0: control, S0C1: 0.3% caraway oil, S0C2: 0.6% caraway oil, S1C0: 1.5 mM Salicylic acid, S1C1: 1.5 mM Salicylic acid + 0.3% caraway oil, S1C2: 1.5 mM Salicylic acid + 0.6% caraway oil, S2C0: 3 mM Salicylic acid, S2C1: 3 mM Salicylic acid + 0.3% caraway oil, S2C2: 3 mM Salicylic acid + 0.6% caraway oil. Values represent mean ± SD of three replications. Different letters indicate significant differences at the 5% level according to the Duncan test.

Figure 9 depicts the impact of SA foliar spraying and caraway oil coating as natural bio-preservatives on the degradation incidence. With the passing of the storage term for the control and all treated fruit, the incidence of deterioration on sweet pepper fruit rose. The lowest decay incidence of pepper fruit was below 20% in S2C2 treatment, followed by S0C2 treatment (25%); in contrast, the control pepper fruit showed the highest decay incidence (90%) at the end of the storage period (Figure 9).

According to the results obtained from antimicrobial characteristics of caraway oil (Seidler-Łozykowska et al., 2013), treated fruits might be protected against pathogen attack, and the decay incidence of these fruits reduced. Our results are in line with the findings of Xing et al. (2011) and Ullah et al. (2017) who reported a diminished decay incidence in cinnamon oil-coated sweet pepper for 35 d and bell pepper for 24 d at 8°C, respectively. In general, essential oils can inhibit the fungal infection through the disruption of the fungal membrane integrity and, thereby, inhibit respiration and ion transport processes (Knobloch et al., 1989). The ability of exogenous application of SA in postharvest decay control on numerous horticultural crops was reported by Asghari and Aghdam (2010). SA is a major component in the signal transduction pathway of plants playing an important role in disease resistance (Asghari and Aghdam, 2010). Our results showed that SA foliar spraying incorporated with caraway oil coating significantly increased longevity of sweet pepper fruit under 4 ± 2°C by 60 d, and decay incidence diminished at S2C2 treatment by 5-fold compared to the control fruit.

### Capsaicin content

**Figure 10:**
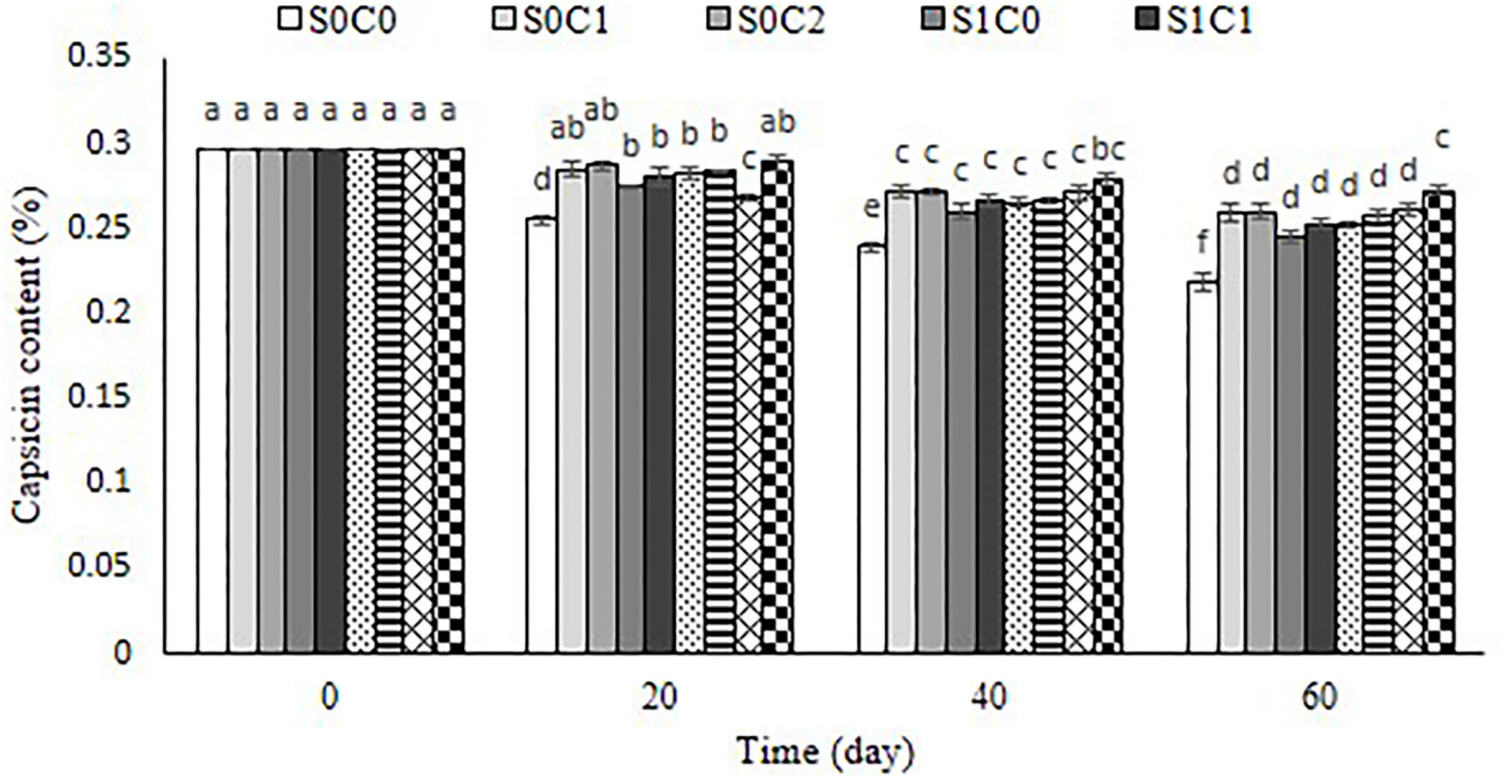
Effect of foliar spraying of SA and caraway oil coating on capsaicin content (%) during 60 d of storage at 4 ± 2°C plus shelf life (2 d, 20°C). S0C0: control, S0C1: 0.3% caraway oil, S0C2: 0.6% caraway oil, S1C0: 1.5 mM Salicylic acid, S1C1: 1.5 mM Salicylic acid + 0.3% caraway oil, S1C2: 1.5 mM Salicylic acid + 0.6% caraway oil, S2C0: 3 mM Salicylic acid, S2C1: 3 mM Salicylic acid + 0.3% caraway oil, S2C2: 3 mM Salicylic acid + 0.6% caraway oil. Values represent mean ± SD of three replications. Different letters indicate significant differences at the 5% level according to the Duncan test.

The condensation of vanillyl amine with short-chain branched fatty acids leads to capsaicinoids accumulation in pepper fruit. The capsicum content in pepper fruit is connected to capsicum's strong taste. In the current investigation, the capsaicin level of untreated and treated fruit decreased as storage time passed (Figure 10). SA foliar spraying incorporated with caraway oil coating in high concentration significantly preserved capsaicin content to a better extent than other treatments. However, its lowest content was observed in the control fruit. Previous studies confirmed that capsaicinoids can be affected by fruit size, age, nutrition, and storage development (Estrada et al., 2002). The degradation of capsaicin into secondary products could possibly be due to the accumulation of reactive oxygen species during the storage period in fruit (Patel et al., 2019). A higher induction of antioxidant enzyme activity following SA caraway oil application led to the radical scavenging potential in treated pepper fruit. Our results are in accordance with the earlier observations, which showed a significant retention of capsaicin in the spermidine- and putrescine-treated capsicum fruits (Patel et al., 2019).

### Correlation study

Correlation analysis (Table 2) reflected significant positive Pearson correlations between CI and weight loss, TSS, pH, chroma, ΔE, MDA content, membrane leakage, total phenol, SOD, and decay incidence during storage at low temperatures, while a significant negative Pearson correlation was observed between CI and firmness, TA and capsaicin content. Decay incidence showed positive and significant correlation with weight loss, TSS, MDA content, and membrane leakage, but has a significant negative correlation with firmness and TA. Capsaicin content showed a significant negative correlation with weight loss, TSS, MDA content, and membrane leakage, while it showed a significant positive correlation with firmness and TA. Our correlation analysis indicated that the reduction of chilling injury in treated fruits can be related to the decrease in the changes in physicochemical properties, and SA foliar spraying incorporated with caraway oil coating has a key role to minimize the decay percentage and maintain the highest level of the bioactive component. In a similar context, SA increased the antioxidant enzymes activity of mandarin fruit under cold storage and reduced the decay percentage (Haider et al., 2020).

**Table 2:** Pearson correlation matrix for chilling injury and physiological properties (weight loss, firmness, and color), biochemical properties (TSS, TA, pH, MDA content, membrane leakage, total phenol, and capsaicin content), decay incidence, and antioxidant enzymes activity (CAT, POD, and SOD) in Sweet pepper fruit treated with foliar spraying of SA and caraway oil-coating after 60 d of storage period at 4 ± 2°C.

	**CI**	**Weight loss**	**Firmness**	**TSS**	**TA**	**pH**	**L**	**Chroma**	**ΔE**	**MDA content**	**electrolyte** **leakage**	**Total phenol**	**CAT**	**POD**	**SOD**	**Decay incidence**
Weight loss	0.965**															
Firmness	−0.872**	−0.869**														
TSS	0.810**	0.883**	−0.925**													
TA	−0.749**	−0.826**	0.946**	−0.905**												
pH	0.712**	0.746**	−0.840**	0.811**	−0.867**											
L	−0.799**	−0.799**	0.941**	−0.0888**	0.958**	−0.811**										
Chroma	0.892**	0.892**	−0.863**	0.829**	−0.853**	0.762**	−0.843 **									
ΔE	0.908**	0.908**	−0.943**	0.888**	−0.904**	0.779**	−0.934**	0.934**								
MDA content	0.867**	0.918**	−0.953**	0.904**	−0.930**	0.794**	−0.0932** 0.925**	0.978**								
electrolyte leakage	0.918**	0.949**	−0.946**	0.989**	−0.878**	0.763**	−0.910**	0.929**	0.987**	0.973**						
Total phenolic	0.877**	0.914**	−0.962**	0.912**	−0.915**	0.793**	−0.942**	0.902**	0.982**	0.977**	0.986**					
CAT	−0.231*	−0.299**	−0.069^ns^	0.050^ns^	0.038^ns^	0.017^ns^	0.066^ns^	−0.346**	−0.174^ns^	−0.130^ns^	−0.122^ns^	−0.064^ns^				
POD	0.39^ns^	0.175^ns^	−0.396**	0.373**	−0.565**	0.459**	−0.410**	0.299**	0.223*	0.313**	0.188^ns^	0.250**	0.156^ns^			
SOD	0.191*	−0.182^ns^	0.01^ns^	−0.84^ns^	−0.177^ns^	0.020^ns^	−0.0211*	−0.062^ns^	0.03^ns^	0.028^ns^	−0.063^ns^	−0.005^ns^	−0.339**	0.267**		
Decay incidence	0.909**	0.850**	−0.768**	0.772**	−0.693**	0.685**	−0.775**	0.788**	0.846**	0.804**	0.842**	0.822**	−0.187^ns^	−0.076^ns^	−0.150^ns^	
Capsaicin content	−0.810**	−0.801**	0.822**	−0.831**	0.798**	−0.739**	0.832**	−0.780**	−0.852**	−0.831**	−0.838**	−0.843**	0.079^ns^	−0.0147^ns^	0.064^ns^	−0.874**

** and * indicate significantly of correlation at the 1 and 5%, respectively. ns indicate insignificantly of correlation.

## Conclusion

CI of sweet pepper fruit during storage at low temperatures decreases this product's postharvest quality, marketability, and economic losses. The effect of practical use of healthy and natural materials on alleviating the CI and microbial spoilage of sweet pepper fruit during 60 d of cold storage showed that using Salicylic acid as foliar spraying close to harvest stage incorporated with postharvest caraway oil coating alleviated CI and reduced weight loss. We propose utilizing SA (3 mM) and caraway oil essential (0.6%) to preserve the quality of sweet pepper fruit for 60 d of cold storage. In addition, their application resulted in a substantial retention of firmness and biochemical features, high level of antioxidant enzyme activity, and a low incidence of decay in the treated fruits to the end of the experiment. According to the results, we recommend using SA (3 mM) and caraway oil essential (0.6%) to maintain the quality of sweet pepper fruit during 60 d at cold storage. Therefore, these treatments can be considered an industrial method to increase longevity, maintain the quality, and marketability of sweet pepper.

## Data availability statement

The raw data supporting the conclusions of this article will be made available by the authors, without undue reservation.

## Author contributions

SH contributed to pepper greenhouse culture establishment, pre- and postharvest plant treatment, and physicochemical traits measurement and enzyme activity assay. HB conceived and designed research, supervised the whole experiments, wrote the manuscript, and carried out all data of this manuscript analysis. ZG prepared the figures and revised the manuscript. All authors read and approved the final manuscript.

## Conflict of interest

The authors declare that the research was conducted in the absence of any commercial or financial relationships that could be construed as a potential conflict of interest.

## Publisher's note

All claims expressed in this article are solely those of the authors and do not necessarily represent those of their affiliated organizations, or those of the publisher, the editors and the reviewers. Any product that may be evaluated in this article, or claim that may be made by its manufacturer, is not guaranteed or endorsed by the publisher.
